# The jasmonate-responsive GTR1 transporter is required for gibberellin-mediated stamen development in *Arabidopsis*

**DOI:** 10.1038/ncomms7095

**Published:** 2015-02-04

**Authors:** Hikaru Saito, Takaya Oikawa, Shin Hamamoto, Yasuhiro Ishimaru, Miyu Kanamori-Sato, Yuko Sasaki-Sekimoto, Tomoya Utsumi, Jing Chen, Yuri Kanno, Shinji Masuda, Yuji Kamiya, Mitsunori Seo, Nobuyuki Uozumi, Minoru Ueda, Hiroyuki Ohta

**Affiliations:** 1 Graduate School of Bioscience and Biotechnology, Tokyo Institute of Technology, 4259-B65 Nagatsuta-cho Midori-ku, Yokohama 226-8501, Japan; 2 Graduate School of Science, Tohoku University, 6-3, Aramaki-Aza-Aoba, Aoba-ku, Sendai 980-0845, Japan; 3 Graduate School of Engineering, Tohoku University, 6-6-07, Aobayama, Aoba-ku, Sendai 980-8579, Japan; 4 Earth-Life Science Institute, Tokyo Institute of Technology, 2-12-1-IE-1 Ookayama, Meguro-ku, Tokyo 152-8551, Japan; 5 RIKEN Center for Sustainable Resource Science, 1-7-22 Suehiro-cho Tsurumi-ku, Yokohama 230-0045, Japan; 6 Center for Biological Resources and Informatics, Tokyo Institute of Technology, 4259-B65 Nagatsuta-cho Midori-ku, Yokohama 226-8501, Japan

## Abstract

Plant hormones are transported across cell membranes during various physiological events. Recent identification of abscisic acid and strigolactone transporters suggests that transport of various plant hormones across membranes does not occur by simple diffusion but requires transporter proteins that are strictly regulated during development. Here, we report that a major glucosinolate transporter, GTR1/NPF2.10, is multifunctional and may be involved in hormone transport in *Arabidopsis thaliana*. When heterologously expressed in oocytes, GTR1 transports jasmonoyl-isoleucine and gibberellin in addition to glucosinolates. *gtr1* mutants are severely impaired in filament elongation and anther dehiscence resulting in reduced fertility, but these phenotypes can be rescued by gibberellin treatment. These results suggest that GTR1 may be a multifunctional transporter for the structurally distinct compounds glucosinolates, jasmonoyl-isoleucine and gibberellin, and may positively regulate stamen development by mediating gibberellin supply.

## Abstract (web summary)

GTR1 is known to transport glucosinolates in *Arabidopsis*. Here, Saito *et al*. show that GTR1 also transports the plant hormones jasmonate and gibberellin when heterologously expressed in *Xenopus* oocytes, and that *gtr1* mutant plants show a gibberellin-related fertility phenotype.

## Graphical abstract

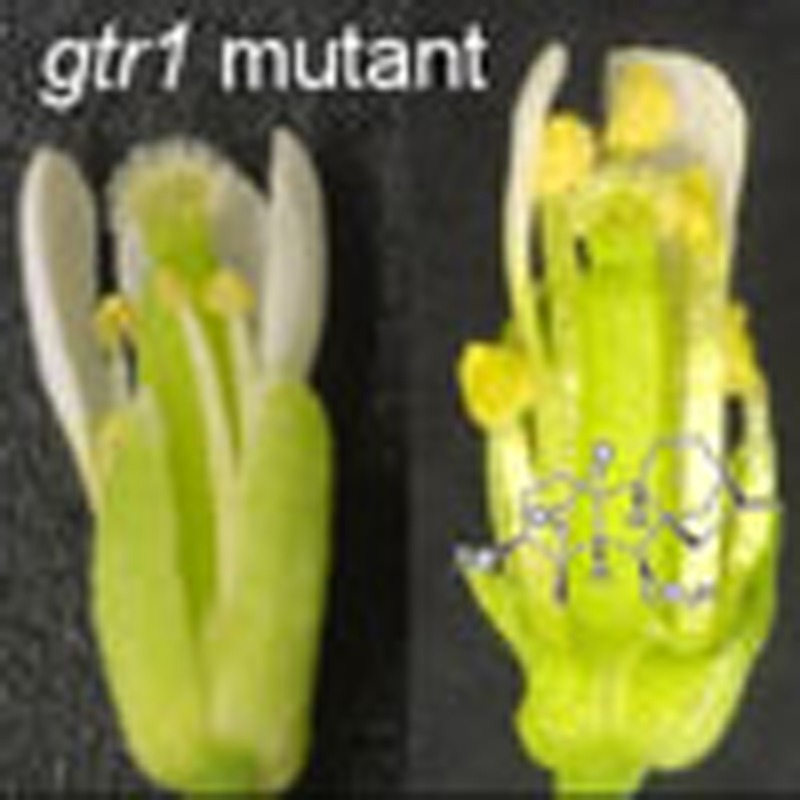

Plant hormones are signalling molecules that induce a wide spectrum of physiological responses at extremely low concentrations. Both import and export of plant hormones across the plasma membrane are important steps preceding intra- and extracellular responses. Hormone transport across the plasma membrane does not take place by simple diffusion but generally requires transporter proteins. In plants, hormone transport is important for controlling various physiological responses. Recent studies have identified several hormone-transporting proteins. Auxin transporters have been identified by mutant analysis based on abnormal phenotypes in their organ development or responses to exogenous auxins or environmental stimuli^1^. Another study identified a different type of auxin transporter, NRT1.1/NPF6.3, which belongs to the NITRATE TRANSPORTER 1/PEPTIDE TRANSPORTER FAMILY (NPF)^2^,^3^. NRT1.1/NPF6.3 facilitates the uptake of auxin, and nitrate inhibits this uptake^3^. Two ATP-binding cassette (ABC)-type transporters were identified as abscisic acid (ABA) transporters; AtABCG25 mediates ABA export from cells, and AtABCG40 mediates ABA uptake^4^,^5^.

A modified yeast two-hybrid system was used to identify another type of ABA transporter, AIT1/NPF4.6, which also belongs to the NPF family^4^. AIT1/NPF4.6 mutants are less sensitive to exogenously applied ABA during seed germination and/or post-germination growth^6^. A DTX/multidrug and toxic compound extrusion family member; AtDTX50 functions as an ABA efflux transporter^7^. An ATP-binding cassette transporter in *Arabidopsis*, AtABCG14, is essential for the translocation of the root-synthesized cytokinins^8^,^9^. The ABC transporter PDR1 in *Petunia hybrida* was identified as a key regulator in the initiation of arbuscular mycorrhizae and axillary branches by functioning as a cellular strigolactone exporter^10^.

Movement of gibberellin (GA) and jasmonates (JAs) may also be important in various physiological events including seed germination, flower induction and development^11^,^12^,^13^,^14^,^15^,^16^,^17^,^18^. However, transporters for these hormones have not been identified except for the *in vitro* GA transport activity of AIT3/NPF4.1 (ref. 6). In plants, JAs are α-linolenic acid-derived hormones that regulate a wide spectrum of plant processes such as pollen and stamen maturation, senescence and defence responses against biotic and abiotic stresses^14^,^15^,^16^. In particular, jasmonoyl-isoleucine (JA-Ile) is a bioactive form of JA that is involved in various physiological responses^14^. JA-deficient *Arabidopsis* mutants are male sterile because of defects in filament elongation, completion of anthesis and anther dehiscence^17^,^18^. Stamen development is also regulated by GA, which is essential for not only floral development but also seed germination, shoot and root growth, and fruit and seed development^11^,^12^,^13^. The GA-deficient mutant *ga1-3* is a male sterile dwarf, and the phenotype can be converted to the wild type (WT) by repeated application of GA^19^,^20^,^21^. Anatomical analysis of this mutant revealed that the male sterile phenotype is because of the arrest of stamen filament cell elongation and failure to complete anthesis^22^. These findings indicate the importance of JA and GA in flower and seed development.

Recently, some details of JA–GA crosstalk have emerged, and the interplay between these two hormones regulates growth and defence^23^,^24^,^25^. DELLA proteins, which are negative regulators of GA signalling, directly interact with JAZ (jasmonate ZIM-domain) repressors of the JA-responsive transcription factor MYC2, suggesting that DELLAs compete with MYC2 for binding to JAZ and modulate JA signalling^23^. Another study showed that sesquiterpene synthase genes are synergistically induced by JA and GA in inflorescences. DELLAs directly interact with MYC2, which promotes the transcription of genes encoding sesquiterpene biosynthetic genes, suggesting that JA and GA promote sesquiterpene biosynthesis via degradation of JAZs and DELLAs, respectively, which are negative regulators of MYC2 (ref. 24). Both JA and GA are known to be important for various developmental processes and accumulating evidence indicates the existence of JA–GA crosstalk in their signalling. However, functional interplay of JA and GA in a specific developmental stage is still largely unknown.

Gene network analysis is an effective way to find sets of genes, which are functionally correlated in some signalling processes^26^. Here we identified the JA-responsive NPF family protein, GTR1/NPF2.10, using co-expression analysis of JA-responsive genes. The GTR1 protein showed GA and JA-Ile transport activities, and the *gtr1* mutant showed decreased fertility owing to impaired stamen development. Supplementation with bioactive GAs at the flowering stage, however, fully restored the phenotype to that of the wild type (WT). These results suggest that GTR1/NPF2.10 may be involved in stamen development via active GA supply.

## Results

### Identification of GTR1

To identify a novel regulator(s) of JA signalling, we searched co-expressed genes of nine jasmonate biosynthetic genes (*DAD1*, *LOX3*, *LOX4*, *AOS*, *AOC3*, *AOC4*, *OPR3*, *OPCL1* and *JAR1*) using ATTED-II CoExSearch ver. c4.1. (http://atted.jp)^27^. We selected genes, which co-expressed with JA biosynthetic genes in the top 20 mutual rank of the Pearson’s correlation coefficient^28^ (Supplementary table 1). We found several genes, as well as *JAZ* gene family and transcription factors, simultaneously expressed with JA biosynthetic genes. A unique NPF family gene, *AT3G47960/GTR1/NPF2.10*, which encodes a glucosinolate transporter^29^, was tightly co-expressed with JA biosynthesis genes such as *LOX3*, *AOS*, *OPR3* and *JAR1*. A recent study on NRT1.1/NPF6.3, which belongs to the same transporter family as GTR1/NPF2.10, showed that the protein is an auxin transporter^3^. Another study on NPF family showed that AIT1/NPF4.6 is an ABA transporter^6^. This study also showed that another NPF protein, AIT3/NPF4.1, has ABA and GA transport activities, suggesting that GTR1/NPF2.10 may transport JAs or other hormones.

### GTR1 is JA-responsive and regulates JA-related genes

**Figure 1:**
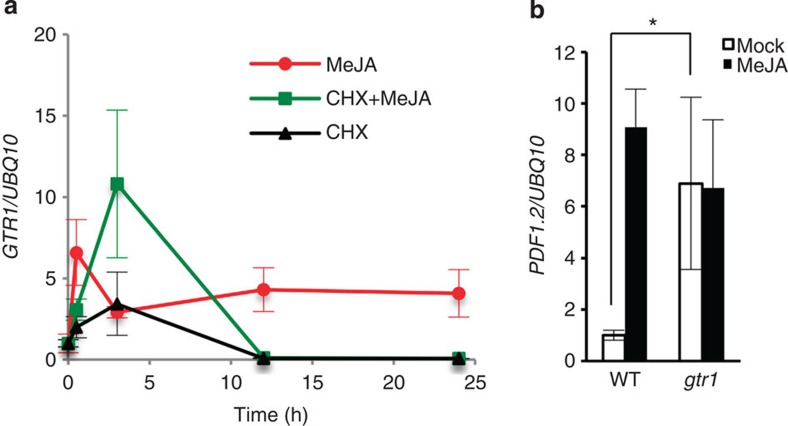
*GTR1* is induced by JA and regulates a JA-related gene. (**a**) Effect of cycloheximide (CHX) on MeJA induction. Ten-day-old liquid-cultured *Arabidopsis* Columbia WT was treated with or without 100 μM CHX for 1 h before treatment with or without 20 μM MeJA for the number of hours indicated, followed by quantitative RT–PCR of *GTR1* transcript levels. *UBQ10* was used as a reference gene. Relative gene expression was calculated by normalizing to the value of mock-treated plants (0.02% ethanol) for 0 h. Values are the mean±s.d. of three biological replicates. (**b**) Expression of *PDF1.2* in *gtr1* seedlings. Ten-day-old liquid-cultured *Arabidopsis* Columbia WT or *gtr1* seedlings were treated with 0.02% ethanol (white bars) or 20 μM MeJA (black bars) for 24 h, followed by quantitative RT–PCR. *UBQ10* was used as a reference gene. Relative gene expression was calculated by normalizing to the value of WT plants treated with 0.02% ethanol. Values are the mean±s.d. of three biological replicates. *P<0.05; Tukey–Kramer comparison test.

To analyse the response of *GTR1* to JAs in detail, we treated liquid-cultured 10-day-old *Arabidopsis* seedlings with 20 μM methyl jasmonate (MeJA) and then analysed *GTR1* transcript levels with quantitative PCR with reverse transcription (RT–PCR). A previous study indicated that *MYC2* and JAZ family genes are rapidly induced by MeJA^30^,^31^,^32^,^33^. *GTR1* was increased in response to MeJA in 30 min, and high expression was maintained for 24 h after treatment (Fig. 1a). We also analysed *GTR1* expression upon cycloheximide (CHX) treatment to test whether the expression requires protein synthesis. *GTR1* was upregulated more so and at a later time in the presence of CHX+MeJA compared with single treatment with MeJA, and *GTR1* expression also increased upon treatment with CHX alone (Fig. 1a). These data suggest that the transient *GTR1* expression is controlled by a primary responsive repressor whose synthesis is blocked by CHX, resulting in prolonged *GTR1* induction. Next, to elucidate functions of GTR1 in JA responses, we obtained T-DNA insertion lines and confirmed the insertion sites with PCR and sequence analyses (Supplementary Fig. 1a). We did not observe expression of the *GTR1* coding region in the *gtr1* mutant (Supplementary Fig. 1b). Thus, we considered *gtr1* to be a knockout mutant.

To clarify *GTR1* function in JA signalling, we analysed expression of JA-responsive genes. In *gtr1* mutants, *PDF1.2*, a marker gene for jasmonate responses, was highly expressed in the absence of MeJA and did not respond to MeJA treatment for 24 h and kept higher expression level at 24 h after MeJA treatment, whereas *PDF1.2* was induced by MeJA in WT plants (Fig. 1b). *MYC2* expression in the mock-treated *gtr1* mutant was about half that of WT, but expression of other JA-responsive genes including *VSP1*, *ERF1* and *ORA59* did not differ between WT and the *gtr1* mutant (Supplementary Fig. 2). We also analysed the expression of JA biosynthesis genes in *gtr1*, and those levels were almost similar to WT except for *LOX3* and *JMT* (Supplementary Fig. 3). These data suggest that a portion of JA signalling is disrupted in the *gtr1* mutant.

### The *gtr1* mutation affects sensitivity to JA

**Figure 2:**
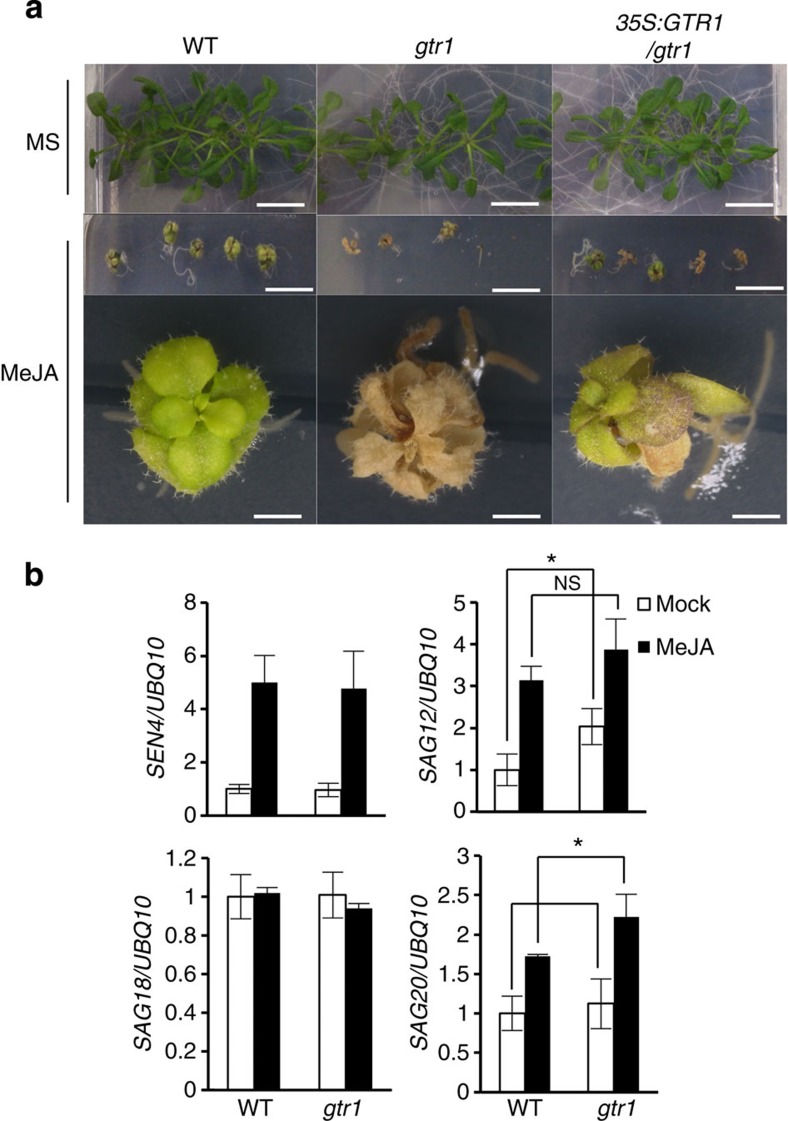
The *gtr1* mutant is hypersensitive to JA. (**a**) Phenotype of 30-day-old seedlings grown on Murashige and Skoog (MS) medium without (top panels) or with (middle panels) MeJA (50 μM). The bottom panels show enlarged images of the seedlings in the second row. Scale bar, 10 mm (top and middle panels), 1 mm (bottom panels). (**b**) Expression of senescence-associated genes in *gtr1* seedlings. Ten-day-old liquid-cultured *Arabidopsis* Columbia WT or *gtr1* seedlings were treated with 0.02% ethanol (white bars) or 20 μM MeJA (black bars) for 72 h, followed by quantitative RT–PCR. *UBQ10* was used as a reference gene. Values are the mean±s.d. of three biological replicates. *P<0.05, NS, not significant (*P*>0.05); Student’s *t*-test.

We created *35S:GTR1/gtr1* lines. Complementation of *GTR1* expression was confirmed with quantitative RT–PCR (Supplementary Fig. 1c). To investigate the sensitivity to MeJA, we grew plants on Murashige and Skoog (MS) medium with or without 50 μM MeJA for 4 weeks. Remarkably, *gtr1* plants turned brown after MeJA treatment, whereas WT plants remained green. *35S:GTR1/gtr1* plants complemented this aspect of the *gtr1* phenotype (Fig. 2a). Plants grown on normal MS medium exhibited no visible differences among WT, *gtr1* and *35S:GTR1/gtr1* plants (Fig. 2a). Next, to clarify whether this de-greening was due to accelerated leaf senescence, we analysed the expression of senescence marker genes^34^ in *gtr1*. *SAG20* was more highly expressed in the MeJA-treated *gtr1* mutant compared with WT, but this was not the case for the mock-treated plants; for *SAG12*, another senescence marker, expression did not differ in the MeJA-treated plants but was significantly higher in mock-treated *gtr1* plants compared with WT (Fig. 2b). The expression levels of *SEN4* and *SAG18* in *gtr1* were comparable to that of MeJA- or mock-treated WT plants, respectively. The upregulated expression of *SAG20* after MeJA treatment suggests that the de-greening phenotype in *gtr1* after MeJA treatment is due to the promotion of JA-induced premature leaf senescence.

### GTR1 positively regulates stamen development

**Figure 3:**
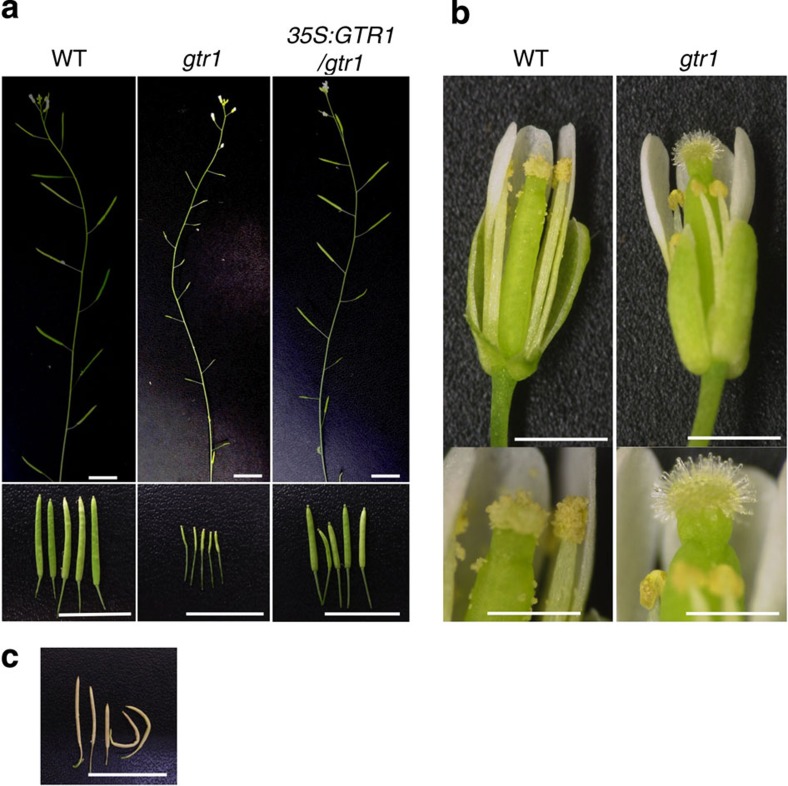
*gtr1* phenotypes in flower. (**a**) The *gtr1* mutant exhibited decreased seed production. The top panel shows representative primary inflorescences. The bottom panel shows representative siliques. Scale bar, 10 mm. (**b**) Phenotypes of flowers. Scale bar, 1 mm (top panel) and 0.5 mm (bottom panel). (**c**) *gtr1* siliques pollinated with WT pollen. Scale bar, 10 mm.

**Figure 4:**
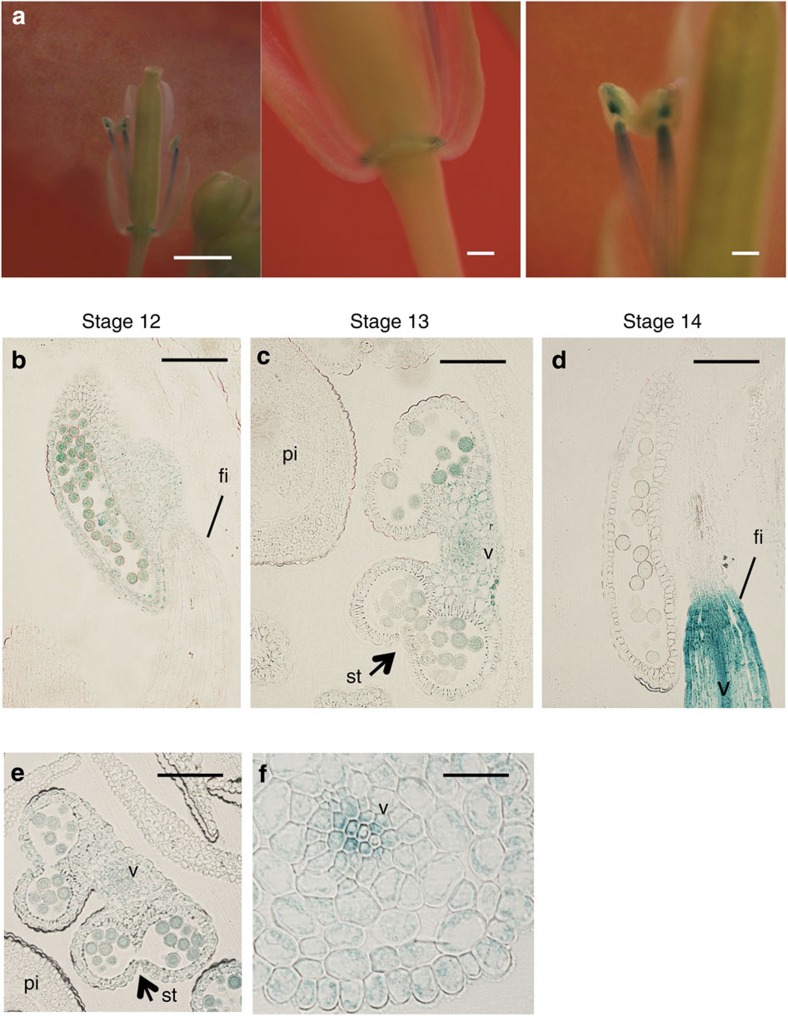
Localization of GTR1 in floral organs. (**a**) Histochemical localization of GUS activity in transgenic plants expressing the *GUS* reporter gene under the control of the *GTR1* promoter. GTR1 was expressed in stamen filaments (left panel), base of filaments (middle panel) and anthers (right panel). Scale bar, 1 mm (left panel) and 100 μm (middle and right panels). (**b**) Vertical section of a *proGTR1::GUS* flower at stage 12. fi, filament. Scale bar, 100 μm. (**c**) Transverse section of a *proGTR1::GUS* flower at stage 13. pi, pistil; st, stomium; v, vascular bundle. Scale bar, 100 μm. (**d**) Vertical section of a *proGTR1::GUS* flower at stage 14. Scale bar, 100 μm. (**e**) Transverse section of a *proGTR1::GUS* flower at stage 12. Scale bar, 100 μm. (**f**) Transverse section of a *proGTR1::GUS* flower at stage 13. Scale bar, 20 μm.

*gtr1* plants have small siliques and reduced fertility, but the relative infertility could be partially reversed upon expression of *35S:GTR1/gtr1* (Fig. 3a). *gtr1* had shorter stamen filaments than the WT and failed in anther dehiscence (Fig. 3b). We also pollinated *gtr1* stigmas with WT pollen (WT*gtr1, Fig. 3c), which yielded mature siliques that were slightly longer than those of WT (12.50±1.56 for WT*gtr1 and 10.50±0.85 for mock-treated WT, error bars are s.d., *n*=10; Table 1), and containing a similar number of seeds compared with WT (34±11.2 for WT*gtr1 and 40.3±5.9 for mock-treated WT, error bars are s.d., *n*=10; Table 2). To test the viability of mature pollen grains, we measured their germination rate; *gtr1* and WT grains had similar germination rates (Supplementary Fig. 4). These data suggest that pollen rarely reached the stigma of *gtr1* plants and that the reduced fertility was caused by a defect in stamen development. Because JA coordinates stamen filament elongation, anther dehiscence and pollen viability at specific stages of flower development^15^,^18^, GTR1 may also regulate stamen development during JA signalling. Indeed, *ProGTR1:β-glucuronidase* (*GUS*) plants exhibited GUS staining in anthers, filaments and the base of filaments, demonstrating the expression of *GTR1* in these floral organs (Fig. 4a).

We observed GUS staining in pollen of stage 12 flowers, but the staining was weak in vascular bundles (Fig. 4b,e). We observed marked GUS staining in vascular bundles of stage 13–14 filaments (Fig. 4c,d,f), at which time filament elongation occurs^35^,^36^.

### GTR1 imports JA-Ile and GA into *Xenopus* oocytes

**Figure 5:**
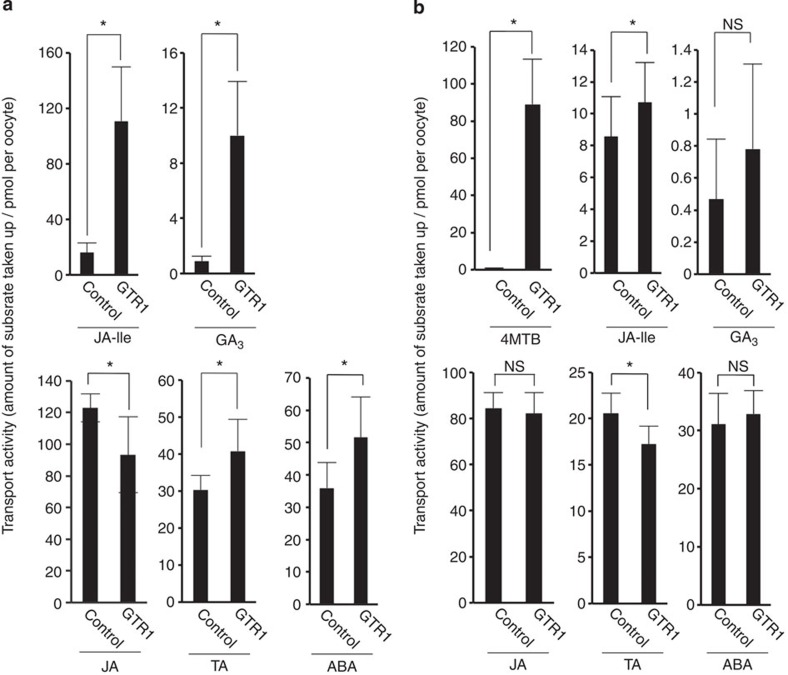
GTR1-mediated transport of hormones into *Xenopus* oocytes. (**a**) GTR1-mediated transport of hormones. Twenty-four hours after cRNA injection, oocytes were added to kulori-based buffer (pH 5.0) containing 100 μM JA, 100 μM TA, 100 μM JA-Ile, 100 μM ABA and 100 μM GA_3_ and incubated at 17 °C for 24 h. Oocytes were washed with sorbitol solution, homogenized in extract medium and incubated at 4 °C for 24 h. After centrifugation, supernatants were collected. Samples were subjected to UPLC/TOFMS analysis. Control means water injection into *Xenopus* oocytes. Values are the mean±s.d. of eight (control) or nine (GTR1) biological replicates. *P<0.05; Student’s *t*-test. (**b**) Activities of GTR1 in the presence of 100 μM 4MTB. Values are the mean±s.d. of 17 biological replicates. *P<0.05, NS, not significant (*P*>0.05); Student’s *t*-test.

Previous studies demonstrated that both GTR1/NPF2.10 and its homologue GTR2/NPF2.11 import glucosinolates, which are major *Arabidopsis* defence compounds, into *Xenopus* oocytes^29^. In addition, the *gtr1gtr2* double mutant does not accumulate glucosinolates in seeds and accumulates excess glucosinolates in source tissues such as leaves and silique walls^29^. However, various *gtr1* phenotypes noted in Figs 1, 2, 3, 4 did not seem to be caused by a defect in glucosinolate transport. A recent study on NRT1.1/NPF6.3, which belongs to the same transporter family (NRT1/PTR Family, NPF) as GTR1/NPF2.10, showed that this protein is an auxin transporter^3^. Another study on AIT1/NPF4.6, which also belongs to the NPF family, showed that this protein is an ABA transporter^6^. That study also showed that another NPF protein, AIT3/NPF4.1, has ABA and GA transport activities^6^, raising the possibility that GTR1/NPF2.10 transports certain phytohormones across the plasma membrane where the protein is located (Supplementary Fig. 5). In addition, *gtr1* showed decreased fertility resulting from a defect in stamen development (Fig. 3b), so we hypothesized that GTR1 could transport JA or GA, which regulate stamen development^11^,^15^,^18^. We therefore assessed the ability of GTR1 to import several plant hormones in *Xenopus* oocytes (Fig. 5). When JA, GA_3_, ABA, JA-Ile or tuberonic acid (TA)^15^ was added to the outside of GTR1-expressing oocytes, GA_3_ and JA-Ile accumulated inside cells, whereas the other phytohormones did not (Fig. 5a). GA_3_ also accumulated in oocytes expressing AIT3/NPF4.1 (Supplementary Fig. 6a), consistent with the report by Kanno *et al*.^6^ Plotting substrate uptake as a function of GA_3_ concentration yielded a saturation curve, which was best fitted by a Michaelis–Menten equation; this yielded an apparent affinity constant of 301.25±225.46 μM (error bars are s.d., *n*=5) for GA_3_ transport by GTR1 (Supplementary Fig. 7). We also analysed GTR1 transport activity in the presence of the phytohormones and 4-methylthiobutyl glucosinolate (4MTB; a glucosinolate with high affinity for GTR1)^29^ and found specific accumulation of 4MTB in GTR1-expressing oocytes (Fig. 5b). These results reveal that GTR1 imports GA_3_ and JA-Ile in cells in the absence of glucosinolates but that 4MTB is preferentially transported compared with GA_3_ and JA-Ile.

### GA rescues the *gtr1* fertility phenotype

**Figure 6:**
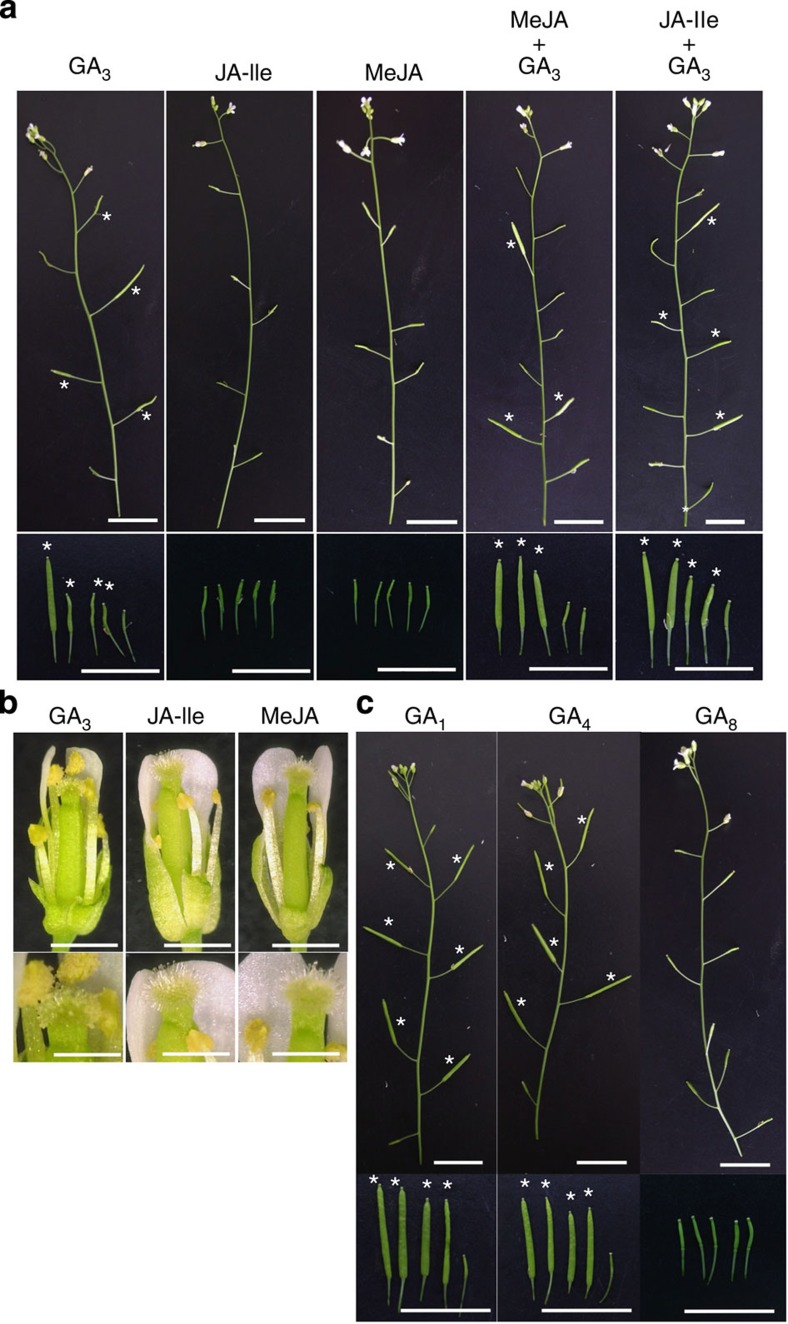
Effects of GAs on decreased fertility in the *gtr1* mutant. (**a**) *gtr1* buds were treated with 50 μM GA_3_, JA-Ile or MeJA as indicated. The top panel shows representative primary inflorescences. The bottom panel shows representative siliques. Stars indicate siliques rescued by hormone treatment. Scale bar, 10 mm. (**b**) Phenotypes of *gtr1* flowers after hormone treatment as indicated. Scale bar, 1 mm (top panel) and 0.5 mm (bottom panel). (**c**) Phenotypes of *gtr1* siliques treated with 50 μM GA_1,_ GA_4_ and GA_8_ as indicated. The top panel shows representative primary inflorescences. The bottom panel shows representative siliques. Stars indicate siliques rescued by hormone treatment. Scale bar, 10 mm.

We hypothesized that GTR1 transports active GA(s) and jasmonate(s) during stamen development. However, the true substrate(s) of the transporter at this developmental stage remained uncertain. We attempted to complement the defect in fertility by treating the flower buds with 50 μM GA_3_, JA-Ile or MeJA. Neither treatment with JA-Ile nor MeJA rescued the defect in fertility. On the other hand, GA_3_ treatment clearly promoted the growth of siliques, resulting in rescue of the decreased fertility (Fig. 6a). We also treated *gtr1* buds with two combinations of hormones (MeJA+GA_3_ or JA-Ile+GA_3_) but observed no statistically significant difference in silique length compared with GA_3_ treatment (Table 1). MeJA+GA_3_ or JA-Ile+GA_3_ treatment increased the seed number of *gtr1* compared with GA_3_ treatment (Table 2). These results indicate that GA restores filament elongation and anther dehiscence and that neither JA-Ile nor MeJA alone appreciably affect those phenotypes in *gtr1* (Fig. 6b).

### Specificity for GAs and the relationship between GA and JA

We analysed the specificity of GTR1 for bioactive and inactive GAs. We attempted to complement the defect in fertility by treating flower buds with 50 μM GA_1_ and GA_4_, which are the bioactive GAs in *Arabidopsis*, or GA_8_, an inactive GA as a negative control. Both GA_1_ and GA_4_ rescued the fertility defect (Fig. 6c). We also analysed the specificity of GTR1 transport activity for bioactive and inactive GAs in *Xenopus* oocytes. GTR1 was capable of transporting GA_3_ but no significant transport of GA_1_, GA_4_, GA_9_ or GA_20_ was observed (Fig. 5 and Supplementary Fig. 6). We measured the content of GA_1_ and GA_4_ in flowers at stages 12, 13 and 14. Each of GA_1_ and GA_4_ was reduced during flower development in both WT and *gtr1*, but the absolute content of each in *gtr1* was significantly lower than that of WT (Supplementary Fig. 8). As a result, we hypothesize that transport of a bioactive GA(s) is impaired in *gtr1*.

**Figure 7:**
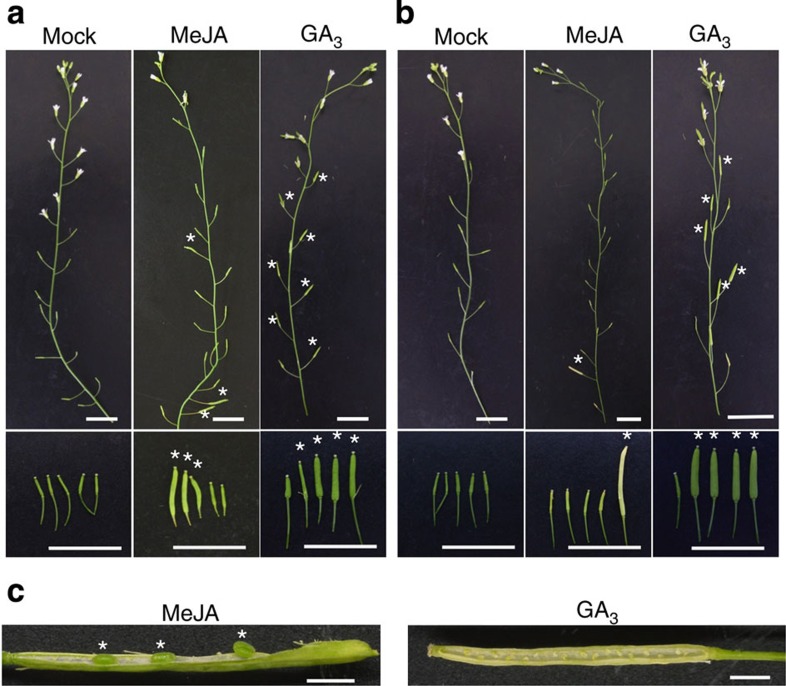
Effects of GA on JA biosynthesis mutants. (**a**) *aos* mutant buds were treated with 0.02% ethanol (mock), 50 μM MeJA or 50 μM GA_3_ as indicated. The top panel shows representative primary inflorescences. The bottom panel shows representative siliques. Stars indicate siliques rescued by hormone treatment. Scale bar, 10 mm. (**b**) *opr3* mutant buds were treated with 0.02% ethanol (mock), 50 μM MeJA or 50 μM GA_3_ as indicated. The top panel shows representative primary inflorescences. The bottom panel shows representative siliques. Stars indicate siliques rescued by hormone treatment. Scale bar, 10 mm. (**c**) *aos* siliques rescued by hormone treatment. Stars indicate rescued seeds. Scale bar, 1 mm.

Early induction of *GTR1* expression by MeJA (Fig. 1) implies that JA is upstream of GA in stamen development. To clarify the relationship between GA and JA in this developmental stage, we attempted to complement the male sterile phenotype of the JA-deficient mutants *aos* and *opr3* with GA_3_. The male sterility of *aos* and *opr3* is rescued by MeJA treatment^18^. *aos* and *opr3* siliques became swollen following GA_3_ treatment, but no mature seeds were found in the siliques (Fig. 7). These results indicated that JA signalling is required for stamen development and is independent of the stimulation of GA signalling, even though GA could partially compensate for a lack of JA function during floral development.

## Discussion

Here we revealed that *gtr1* is a knockout mutant of GTR1/NPF2.10 and shows various phenotypes related to JA and GA function that are especially involved in stamen development. Based on the evidence thus far, we speculate that GTR1 positively regulates filament elongation and anther dehiscence by transporting physiologically important compounds that induce stamen development. GTR1 has been primarily identified as a glucosinolate transporter^29^. Nevertheless, our data showed that 4MTB treatment of *gtr1* buds could not rescue the reduced fertility phenotype (Supplementary Tables 2 and 3). In addition, treatment with 4MTB did not potentiate the effect of GA_3_ and MeJA to restore the fertility of *gtr1* (Tables 1 and 2, Supplementary Tables 2 and 3). These data could indicate that the phenotypes of *gtr1* stamens may not result from a lack of glucosinolate transport but rather from a lack of hormone transport.

Although we analysed bioactive and inactive forms of JAs, only the bioactive jasmonate JA-Ile was transported into the *Xenopus* oocytes by GTR1 (Fig. 5a). However, JA-Ile treatment of *gtr1* buds did not rescue the reduced fertility phenotype (Fig. 6a). GTR1 is capable of transporting only GA_3_ in *Xenopus* oocytes, an active form of gibberellin (Supplementary Fig. 6b), and the other bioactive gibberellins (GA_1_, GA_4_) were able to rescue the *gtr1* phenotype in addition to GA_3_ (Fig. 6). In particular, GA_4_ fully complemented the infertility phenotype (Tables 1 and 2). The *in vivo* substrate for GTR1 remains unclear because we found that only GA_3_ was significantly transported by GTR1 in *Xenopus* oocytes; GA_4_ uptake was observed in oocytes even in the absence of GTR1 expression (Supplementary Fig. 6b), which may be a consequence of simple diffusion into oocytes because GA_4_ is relatively hydrophobic compared with other active GAs. Thus, greater passive diffusion may have mimicked transporter-mediated GA_4_ uptake in oocytes. Recent reports have indicated that posttranslational modification alters a nitrate transporter’s affinity for substrates^37^. Such modifications might regulate the *in vivo* substrate selectivity of GTR1. Despite the ability of GTR1 to transport GA_3_ and JA-lle in oocytes, this does not constitute absolute proof that this is the reason for the *gtr1* fertility phenotype. Therefore the *in vivo* substrate for GTR1 during flower development should be clarified in further work.

Synthesis of bioactive GAs occurs throughout floral bud development^36^. After the establishment of floral organs by stage 7, *GA3ox*, a gene crucial for the synthesis of bioactive GAs, is expressed in the stamen^36^. Very high levels of bioactive GAs have been measured in rice anthers but not in other floral tissues^38^,^39^, suggesting that anther tissues (specifically the tapetum) act as the source of GAs in flowers. Our results show that GA_1_ and GA_4_ contents were reduced from stage 12 to stage 14 in flower development both in WT and *gtr1* (Supplementary Fig. 8). As GA inactivation pathways are also tightly regulated by developmental signals^40^, the accumulated bioactive GAs in anthers might be inactivated in these stages, resulting in reduction of the GAs in whole flowers. As *GTR1* expressed in the stage 14 filaments (Fig. 4), GTR1 could transport bioactive GAs from anthers to the other floral organs such as filaments. We speculate that transportation of the bioactive GAs is impaired in *gtr1* and the GAs are more quickly inactivated in the *gtr1* anther, resulting in more strongly reduced bioactive GA contents compared with WT even in whole flowers (Supplementary Fig. 8).

We consider GA_4_ (and GA_1_) may be a substrate of GTR1 in *Arabidopsis*, and that the decreased supply of these compounds to their principal site of action (perhaps the stamen filaments of stages 13 and 14, as indicated in Fig. 4) may underlie the observed defect in filament elongation. However, detailed localization of bioactive GAs during flower development should be clarified in future.

Because GA_1_ and GA_4_ are the major active GAs in *Arabidopsis*^36^, we suggest the phenotypes observed in *gtr1* stamens may be caused by specific impairment of the supply of bioactive GA_1_ and GA_4_ to such a source tissue. GTR1 transported active GAs in oocytes, but the phenotype of *gtr1* other than that related to fertility was similar to that of WT, suggesting that other GA transporters may exist. GTR2, a close homologue of GTR1, also transported GA_3_ into oocytes (Supplementary Fig. 9). This result together with the high GA transport activity of AIT3/NPF4.1 suggests that several NPF proteins^2^, including NPF2.11 and NPF4.1, may also be involved in GA transport during other developmental processes, and therefore severe developmental abnormalities are not found in the *gtr1gtr2* double mutant^29^. On the other hand, specific expression of GTR1 in floral tissues (Fig. 4) is associated with the restricted function of GTR1 during stamen development.

The effects of JA and GA on seed number are probably additive (Table 2). GA_3_ treatment of the JA-deficient mutant *aos* and *opr3* induced swelling of siliques, but no mature seeds were produced (Fig. 7a–c). Thus, GA and JA complement each other’s function only partially during floral development, and GA signalling is not simply downstream of JA signalling. Rather, these two signalling pathways function in parallel and coordinate with each other during stamen development. GTR1 is expressed in filaments—especially in vascular bundles—in stages 13 and 14 (Fig. 4d–f), so we suggest that GTR1 may transfer GAs from vascular bundles to cells. Therefore, imported GAs may promote cell expansion to drive filament elongation in stages 13 and 14. On the other hand, GTR1 was expressed less in the tapetum and surrounding tissues that undergo secondary thickening to create the mechanical force necessary for dehiscence. Filament elongation may be a GA-regulated process, whereas anther dehiscence may be primarily JA regulated. On the basis of this evidence, we suggest that GTR1 may promote stamen development mainly by supplying GAs to the stamen filaments.

In addition to its possible role in GA transport, GTR1 transports glucosinolates^29^ and JA-Ile in oocytes (our present study), and thus GTR1 may function during various physiological events. GTR1 may be involved in JA signalling (Figs 1 and 2, Supplementary Figs 2 and 10). The *gtr1* mutation affected root elongation—an indicator of JA responses—in the presence of MeJA (Supplementary Fig. 10). *SAG20* was highly expressed in *gtr1* in response to MeJA (Fig. 2). We speculate that in *gtr1* leaves, GTR1-dependent hormone transport may be impaired, and this may cause stress-associated senescence, resulting in the de-greening phenotype. The reason for *SAG12* induction in untreated *gtr1* leaves is unknown. Dysregulation of endogenous hormone levels in *gtr1* may constitute the molecular basis for senescence.

GA alone induced *PDF1.2* expression in WT plants, but this induction was not observed in *gtr1* (Supplementary Fig. 11). When we compared the effects of the *gtr1* mutation on *PDF1.2* expression following the treatment with GA or MeJA, the expression was higher following GA treatment. We suggest that the compromised *PDF1.2* expression observed in *gtr1* in the absence of GA supplementation may be mainly caused by the disruption of canonical GA transport in leaves and that impaired GA transport may affect the JA-induced senescence phenotype. In fact, the *pif* quadruple mutant, which is defective for GA signalling, shows compromised JA responses^25^, leading us to suggest that JA-hypersensitive *gtr1* phenotypes may be associated with the absence of GA transport, which could affect the crosstalk between the GA and JA signalling networks^23^,^24^,^25^. However, possible GTR1-mediated JA-Ile transport may be of fundamental importance for these JA-related functions. We found that GTR1 was expressed in shoots, roots and around apical meristems (Supplementary Fig. 12), and a previous report showed that GTR1 is expressed in senescent leaves and that glucosinolates, which are principal substrates for GTR1, are transported from senescent leaves to seeds^29^. Therefore, we speculate that GTR1 may regulate senescence processes in leaves by facilitating hormone transport.

We propose that GTR1/NPF2.10 may be a multifunctional transporter with substrates that are diverse in structure and function. We suggest that GTR1 may have a crucial role in supplying GA for stamen development and male fertility, a process that is tightly regulated by both JA and GA. We also propose that substrate distribution is an important factor in the activity of this type of multifunctional transporter and raise the possibility that GTR1 also functions in hormone transport in vegetative tissues.

## Methods

### Quantitative RT–PCR

Total RNA was isolated from the indicated plant materials using the SV Total RNA Isolation System (Promega KK, Tokyo, Japan). Total RNA (400 ng) was converted to cDNA using PrimeScript RT Enzyme Mix I, Oligo dT Primers and Random 6-mers according to the PrimeScript 1st strand cDNA Synthesis kit protocol (TaKaRa, Shiga, Japan). Quantitative RT–PCR was performed with 8.0 ng cDNA in a final volume of 20 μl according to the SYBR Premix Ex Taq II instructions manual (TaKaRa, Shiga, Japan). Quantitative RT–PCR was performed in a Thermal Cycler Dice Real Time System II (TaKaRa, Shiga, Japan). The data were normalized to data for mock-treated WT. The polyubiquitin gene *UBQ10* At4g05320 (ref. 41) was used as a reference gene. The thermal cycling conditions were: initial denaturation at 95 °C for 30 s, followed by 40 cycles of 5 s at 95 °C and 30 s at 60 °C. Three biological replicates were used for each experiment. The primers used were: *UBQ10* (*At4g05320*) forward 5′- GGCCTTGTATAATCCCTGATGAATAAG -3′, reverse 5′- AAAGAGATAACAGGAACGGAAACATAGT -3′; *GTR1* (*At3g47960*) qPCR forward 5′- GTCCATTGGCTGGTATTGCT -3′, reverse 5′- ACTTGCTGCAACGTGCATAG -3′; *PDF1.2* (*AT5G44420*) forward 5′- TTTGCTGCTTTCGACGCAC -3′, reverse 5′- CGCAAACCCCTGACCATG -3′; *MYC2* (*AT1G32640*) forward 5′- CGGCTACAACCAACGATGAA -3′, reverse 5′- CCGGAGGCCATAAAGTTGAG -3′; *ERF1* (*AT3G23240*) forward 5′- ACGATCCCTAACCGAAAACAGA -3′, reverse 5′- GTGAGAAGCCGGAGAATGG -3′; *ORA59* (*AT1G06160*) forward 5′- GGCTCTCGCTTATGATCAGG -3′, reverse 5′- CCGGAGAGATTCTTCAACGA -3′; *LOX3* (*AT1G17420*) forward 5′- CACTGCAATTCACAAGCAACC -3′, reverse 5′- CAAAGGAGGAATCGGAGAAGC -3′; *AOS* (*AT5G42650*) forward 5′- GCGACGAGAGATCCGAAGA -3′, reverse 5′- CTCGCCACCAAAACAACAAA -3′; *DAD1* (*AT2G44810*) forward 5′- GTGAAGACGAAGAAGAAGAGCAATC -3′, reverse 5′- GTGAAGACAGCGAAAACGACATAC -3′; *OPR3* (*AT2G06050*) forward 5′- TTGGACGCAACTGATTCTGAC -3′, reverse 5′- GTAGGCGTGGTAGCGAGGTT -3′; *SEN4* (*AT4G30270*) forward 5′- GACTCTTCTCGTGGCGGCGT -3′, reverse 5′- CCCACGGCCATTTCCCCAAGC -3′; *SAG12* (*AT5G45890*) forward 5′- GGCGTTTTCAGCGGTTGCGG -3′, reverse 5′- CCGCCTTCGCAGCCAAAATCG -3′; *SAG18* (*AT1G71190*) forward 5′- GTTTGCGAGGTGAGAAAATAGGA -3′, reverse 5′- AGAGTAGCATCGTTTGGGTGAAG -3′; *SAG20* (*AT3G10985*) forward 5′- TCGGTAACGTTGTTGCTGGA -3′, reverse 5′- ACCAAACTCTTTCAAATCGCCA -3′.

### Genotyping and expression analysis of T-DNA knockout plants

T-DNA insertion mutants in the Col-0 background were from The Arabidopsis Biological Resource Center: *gtr1*, (CS879742). Genotyping analysis was performed with PCR. Primers used were: *GTR* forward 5′- ATGGAGAGAAAGCCTCTTG -3′, reverse 5′- TCAGACAGAGTTCTTGTCT -3′; *LB3* 5′- TAGCATCTGAATTTCATAACCAATCTCGA -3′. To produce *35S:GTR1/gtr1* plants, the *GTR1* sequence was PCR amplified with PrimeSTAR HS DNA Polymerase (TaKaRa, Shiga, Japan) from cDNA generated using the RNA PCR Kit (TaKaRa, Shiga, Japan) and Gateway-compatible primers (forward 5′- GGGGACAAGTTTGTACAAAAAAGCAGGCTACACC -3′, reverse 5′- GGGGACCACTTTGTACAAGAAAGCTGGGTATCAGACAGAGTTCTTGTCT -3′). PCR products were cloned into pDONR/Zeo with the Gateway BPII kit (Life Technologies, MD, USA), and sequences were verified. The *GTR1* constructs were used in Gateway (Life Technologies, MD, USA) LR reactions in combination with the binary expression vectors pGWB2 (ref. 42) and were then checked by sequencing. The resulting construct was then transformed into *gtr1* using the *Agrobacterium tumefaciens*-mediated floral dip method^43^.

Expression analysis was performed with semi-quantitative RT–PCR. For RT–PCR, total RNA was isolated using the SV Total RNA Isolation System (Promega KK, Tokyo, Japan), cDNA was generated using the RNA PCR kit (TaKaRa, Shiga, Japan) and PCR was performed. *Actin2* (*AT3G18780*) was used as the reference gene^41^. Primers used were: *GTR1* (*AT3G47960*) RT–PCR forward 5′- ATGGAGAGAAAGCCTCTTG -3′, reverse 5′- TCAGACAGAGTTCTTGTCT -3′; *Actin2* (*AT3G18780*) forward 5′- ACATTGTGCTCAGTGGTGGA -3′, reverse 5′- TCATACTCGGCCTTGGAGAT -3′.

### Chemical reagents

TA was synthesized as previously described. (-)-MeJA was prepared from commercially available racemic MeJA according to the previous method^44^,^45^. Ozonolysis of olefin moiety in (-)-MeJA, Wittig reaction and successive deprotection gave TA^46^.

(−)-JA-L-Ile was synthesized according to the previous method^47^. Briefly, racemic MeJA was coupled with L-Ile. After deprotection, the diastereomeric mixture was separated by Silica gel column chromatography to give (−)-JA-L-Ile.

(±)-JA, MeJA, ABA, GA_3_ were purchased from Wako, Osaka, Japan. 4-Methylthiobutylglucosinolate (ChromaDex, CA, USA), GA_1_, GA_4_, GA_8_, GA_9_ and GA_20_ (Olchemim, Olomouc, Czech Republic) were also commercially available.

### Plant growth conditions

For gene expression analyses, we used 10-day-old seedlings. Ten to fifteen seeds were incubated in a 100-ml Erlenmeyer flask with 30 ml liquid MS medium containing 1% sucrose under continuous light (20–36 μmol m^−2^ s^−1^) with shaking (130 r.p.m.). Plants were treated with 20 μM MeJA or/and 20 μM GA_3_ and harvested at the indicated time points. For CHX treatment, plants were treated with 100 μM CHX for 1 h before hormone treatment. Plant materials were frozen in liquid nitrogen and stored at −80 °C until use. To test sensitivity to MeJA, we used 4-week-old seedlings grown on MS medium containing 50 μM MeJA, 0.8% agar and 1% sucrose under continuous light (30–55 μmol m^−2^ s^−1^). We also used the same experimental conditions to measure the root length of plants treated with 20 μM MeJA. For the root length measurement, we placed the plates in the vertical orientation. We photographed the roots and measured the root length with ImageJ software (http://rsbweb.nih.gov/ij)^48^. For adult plants, seedlings grown on MS medium containing 0.8% agar were transferred to the soil and grown to maturity at 22 °C under continuous light. For hormone complementation assays, buds of adult plants were treated with 50 μM GAs, 50 μM MeJA or 50 μM JA-Ile every 2 days for 2 weeks.

### Histochemical analysis

A 2,000-bp, genomic fragment immediately upstream of the *GTR1* start codon was amplified with PCR using primers (forward 5′- TGATTACGCCAAGCTTGGTTCTTAGACTGGCGAG -3′, reverse 5′- ACCTGCAGCCAAGCTTGTCAAGCTTCTCCGCTC -3′) and then cloned into the vector pCambia1391Z. The resulting construct was then transformed into Columbia-0 using the *Agrobacterium tumefaciens*-mediated floral dip method^43^. GUS staining was performed for 16 h with 2 mM X-Gluc (5-bromo-4-chloro-3-indolyl-β-D-glucuronide), 0.5 mM K_4_Fe(CN)_6_, 0.5 mM K_3_Fe(CN)_6_, 0.3% (v/v) Tween20 and 50 mM NaPO_4_, pH 7.4. The reaction was stopped and plants were destained in 70% ethanol^49^. To analyse the hormone responses, plants were treated with 20 μM MeJA or 0.02% ethanol for 3 h before GUS staining. For tissue sectioning, GUS-stained flowers were fixed overnight in FAA solution (5% v/v formalin, 5% v/v acetic acid, 45% v/v ethanol) at 4 °C.

Fixation of the samples was conducted as described^50^ with minor changes. Samples were dehydrated with an ethanol series, and then ethanol was substituted with mixture containing Technovit 7100 solution (Heraeus Kulzer, Tokyo, Japan) and hardener I (Heraeus Kulzer, Tokyo, Japan). Samples were embedded in a mixture containing hardener II solution (Heraeus Kulzer, Tokyo, Japan), Technovit 7100 solution, and hardener I. The samples were sectioned at 6 μm thickness. We photographed the sectioned samples and colour balance of the pictures was adjusted with ImageJ software^48^.

### Characterization of the GTR1 transporter using *Xenopus* oocytes

The *GTR1* coding sequence was amplified with primers (forward 5′- TATTAAGCTTGAATTCATGAAGAGCAGAGTCATTCT -3′, reverse 5′- CGACCCCGGGGAATTCTCAGACAGAGTTCTTGTCT -3′) and subcloned into the *EcoRI* site of the plasmid, which was previously used to express AtHKT1 (ref. 51). *GTR2* was amplified with primers (forward 5′- AAGCTTGAATTCCCCGGGATGGAGAGAAAGCCTCTTG -3′, reverse 5′- GGATCCGTCGACCCCGGGTCAGGCAACGTTCTTGTCT -3′) and subcloned into the *SmaI* site. NPF4.1 was amplified with primers (forward 5′- AAGCTTGAATTCCCCGGGATGCAGATTGAGATGGAAG -3′, reverse 5′- GGATCCGTCGACCCCGGGCTAATATCTTTTCGCCCAG -3′) and subcloned into the *SmaI* site. Capped complementary RNA (cRNA) of each gene was prepared using the mMESSAGE mMACHINE kit (Life Technologies, MD, USA). Oocyte preparation and cRNA injection were performed as described^51^. Each oocyte was injected with 500 ng cRNA. After incubation for 24 or 48 h, the buffer was replaced with 100 μl of kulori-based solution^51^ (90 mM sodium gluconate, 1 mM potassium gluconate, 1 mM calcium gluconate, 1 mM magnesium gluconate, 1 mM LaCl_3_ and 10 mM MES, pH 5) containing the corresponding substrate(s), and oocytes were incubated for an additional 24 h. For Determination of K_m_ values, 24 h after cRNA injection, two oocytes were treated with the GA_3_ substrate (1, 10, 30, 60, 100, 300, 1,000 and 2,000 μM) in a Kulori-based buffer (pH 5) and incubated at 17 °C for 24 h.

### Sample preparation for liquid chromatography/mass spectrometry

Oocytes were washed twice with 200 mM sorbitol solution, homogenized in 40 μl extract buffer (28% methanol, 0.05% acetic acid) and incubated at 4 °C for 24 h. After centrifugation (20,000 *g*, room temperature, 20 min), supernatants were collected, and 10-μl samples were subjected to ultra-performance liquid chromatography coupled to time-of-flight mass spectrometry (UPLC/TOFMS) analysis.

### UPLC/TOFMS analysis

UPLC/TOFMS analysis of 10-μl samples from oocyte extracts was performed using an Agilent 1290 Infinity (Agilent Technologies, CA, USA) coupled to a microTOF II (Bruker Daltonics, Leipzig, Germany). A ZORBAX Eclipse Plus C18 column (1.8 μm, 2.1 × 50 mm; Agilent Technologies) was used to separate substrates. The mobile phases were: A, 20% (v/v) aqueous methanol with 0.05% (v/v) acetic acid, and B, methanol with 0.05% (v/v) acetic acid. The gradient programme was: 0 to 3.5 min, isocratic 90% A; 3.5 to 6 min, linear gradient 90 to 0% A; 6.1 min to 9 min, isocratic 90% A, with a flow rate of 0.15 ml min^−1^. The mass spectrometer was run in the negative mode with scan range 100–700 *m*/*z*. The capillary voltage was 4,200 V, the nebulizer gas pressure was 1.6 bar, the desolvation gas flow was 8.0 l min^−1^, and the temperature was 180 °C. The substrates were quantified on the basis of an extracted ion chromatogram and the corresponding peak position of the standard solution.

### EGFP fusion and subcellular localization

The stop codon of *GTR1* was mutagenized with PCR to introduce a *SalI* site with primers (forward 5′- TACAATTACAGTCGACATGAAGAGCAGAGTCATTCT -3′, reverse 5′- ATCCTCTAGAGTCGACGACAGAGTTCTTGTCT -3′). This site was then used to make an in-frame *GTR1-EGFP* fusion construct. The final construct, 35S:*GTR1-EGFP*-pUC18, and the empty vector, 35S:*EGFP*-pUC18, were transiently expressed in onion epidermal cells using a particle gun-mediated system (PDS-1000/He; Bio-Rad, CA, USA). The bombarded cells were kept in the dark at 22 °C for 12 h followed by GFP imaging using confocal microscopy (Leica; DM2500).

### Pollen germination *in vitro*

The *in vitro* pollen germination experiment was conducted as described^52^ with minor modifications. For WT and *gtr1*, stage 14 flowers were randomly harvested from eight plants each. For each experiment, pollen grains were then obtained from three separate flowers. The medium for pollen germination contained 5 mM MES-Tris (pH 5.8), 1 mM KCl, 0.8 mM MgSO_4_, 1.5 mM boric acid, 10 mM CaCl_2_, 16% (w/v) sucrose and 0.5% (w/v) INA agar. Pollen germination was observed by microscopy after 24 h incubation at 23 °C in continuous light (30 μmol m^−2^ s^−1^) with high humidity, and the number of germinated pollen grains was counted.

### Plant hormone quantification

The developmental stages of flowers were as described^35^. Flowers were categorized into three stages (12–14) and harvested every 3 days from 4- to 6-week-old plants grown on soil. Flower samples (~500 mg fresh weight per stage) were frozen in liquid nitrogen and stored at −80 °C. Frozen samples from WT and *gtr1* were lyophilized, and GAs were extracted from the samples using 80% (v/v) acetone containing 1% (v/v) acetic acid as described^53^. Briefly, lyophilized plant materials (12–28 mg dry weight) were ground in 80% (v/v) acetone containing 1% (v/v) acetic acid with [17,17-^2^H_2_] GAs (300 pg each) as internal standards. Crude extract was purified through polyvinylpyrrolidone (500 mg; Tokyo Kasei) and reverse-phase (Oasis HLB, 60 mg; Waters) cartridges. The GA-containing fraction was further purified by an ion-exchange column (Bond Elut DEA, 100 mg; Agilent) and a SepPak silica cartridge (100 mg; Waters). Obtained GA-enriched fraction was concentrated to dryness, dissolved in 20 μl of water containing 1% acetic acid, and then subjected to LC-tandem mass spectrometry (LC-MS/MS) analysis.

GAs were quantified as described^53^ with some modifications. LC was performed with an Acquity UPLC HSS T3 column (1.8 μm, 2.1 × 50 mm; Waters, MA, USA) using the following programme with solvent A (water containing 0.01% (v/v) acetic acid) and solvent B (acetonitrile containing 0.05% (v/v) acetic acid): isocratic flow with 3% B for 30 s, a linear gradient of B from 3 to 12% over 2.5 min, a linear gradient of B from 12 to 25% over 5 min, a linear gradient of B from 25 to 40% over 4 min, isocratic flow with 40% of B for 2 min, a linear gradient of B from 40 to 98% over 1 min, and isocratic elution with 98% B for 1 min. MS/MS conditions were as follows: ion spray voltage floating (kV)=−4.0, desolvation temperature=600 °C, collision energy (V)=−40 (GA_1_) or −30 (GA_4_), declustering potential=−90, MS/MS transition (*m*/*z*): 349.2/275.2 ([^2^H_2_] GA1), 347.2/273.2 (GA_1_), 333.2/259.2 ([^2^H_2_] GA_4_), 331.2/257.2 (GA_4_). The LC retention time was 5.5 min for GA_1_ and 11.5 min for GA_4_.

## Author contributions

H.S. performed gene expression analyses and subcellular localization experiments. H.S. and M.K.-S. performed physiological analyses and histochemical localization experiments. H.S., T.U., J.C. and M.K.-S. produced transgenic lines. T.O., S.H., and Y.I. of Tohoku University and M.S. of RIKEN characterized the GTR1 transport activity. H.S. and M.K.-S. of Tokyo Institute of Technology and Y. Kanno and M.S. of RIKEN measured the GA content. H.S., Y.S.-S., S.M. and H.O. of Tokyo Institute of Technology, N.U. and M.U. of Tohoku University and M.S. and Y. Kamiya of RIKEN designed most of the experiments and discussed the results. H.S., S.H., Y.I., Y.S.-S., M.K.-S. and H.O. wrote the manuscript. H.O. planned the project. All the authors discussed the results and commented on the manuscript.

## Additional information

**How to cite this article:** Saito, H. *et al*. The jasmonate-responsive GTR1 transporter is required for gibberellin-mediated stamen development in *Arabidopsis*. *Nat. Commun.* 6:6095 doi: 10.1038/ncomms7095 (2015).

## Supplementary Material

Supplementary Information Supplementary Figures 1-12 and Supplementary Tables 1-3

## Figures and Tables

**Table 1:** Effects of hormone treatments on silique lengths.

**Treatment**	**Mock**	**GA**_3_	**MeJA**	**JA-Ile**	**GA**_1_	**GA**_4_	**GA**_8_	**MeJA+GA**_**3**_	**JA-Ile+GA**_3_	**Untreated**
WT	10.50±0.85a	9.30±0.86	9.95±1.28	10.00±0.91	9.25±1.03	9.50±0.97	9.30±1.01	9.75±0.75	9.45±1.04	—
*gtr1*	3.25±0.63b	9.20±1.23a	3.30±1.32	3.75±1.55	8.25±1.4d	11.35±1.18a	3.35±0.85	10.45±1.61a	9.70±1.18a	—
*aos*	2.80±0.26	7.75±1.98	12.05±1.66	—	—	—	—	—	—	—
*opr3*	3.05±0.37	8.65±0.91	10.25±1.78	—	—	—	—	—	—	—
WT * gtr1	—	—	—	—	—	—	—	—	—	12.50±1.56c

Mature silique lengths (mm) were measured. Buds were treated with or without 50 μM GAs or JAs as indicated. Values are the mean±s.d. of 10 biological replicates. Different letters indicate significant differences (*P*<0.05). The data were analysed followed by Tukey–Kramer multiple comparison test. WT * *gtr1* means *gtr1* siliques pollinated with WT pollen.

**Table 2:** Effects of hormone treatments on seed production.

**Treatment**	**Mock**	**GA**_**3**_	**MeJA**	**JA-Ile**	**GA**_**1**_	**GA**_**4**_	**GA**_**8**_	**MeJA+GA**_**3**_	**JA-Ile+GA**_**3**_	**Untreated**
WT	40.3±5.9a	30.5±7.9	29.0±9.7	27.2±8.6	30.8±7.8	28.9±8.4	30.3±5.5	27.9±4.5	27.3±6.1	—
*gtr1*	4.6±4.6b	16.1±7.4b	0.7±2.2b	1.0±1.7b	15.5±8.7b	38.9±11.2a	0.8±1.8b	24.1±11.4c	20.5±6.9c	—
*aos*	0±0	0±0	36.9±11.7	—	—	—	—	—	—	—
*opr3*	0±0	0±0	20.8±10.8	—	—	—	—	—	—	—
WT * gtr1	—	—	—	—	—	—	—	—	—	34±11.2a

The numbers of mature seeds were counted. Buds were treated with or without 50 μM GAs or JAs as indicated. Values are the mean±s.d. of 10 biological replicates. Different letters indicate significant differences (*P*<0.05). The data were analysed followed by Tukey–Kramer multiple comparison test. WT * *gtr1* means *gtr1* seeds pollinated with WT pollen.
