# Supplementary material for: The jasmonate-responsive GTR1 transporter is required for gibberellin-mediated stamen development in Arabidopsis
Source: Nat Commun. 2015 Feb 4;6:6095. doi: 10.1038/ncomms7095 (PMC4347201; doi:10.1038/ncomms7095)
Supplement: Supplementary Information — Supplementary Figures 1-12 and Supplementary Tables 1-3 [file ncomms7095-s1.pdf]

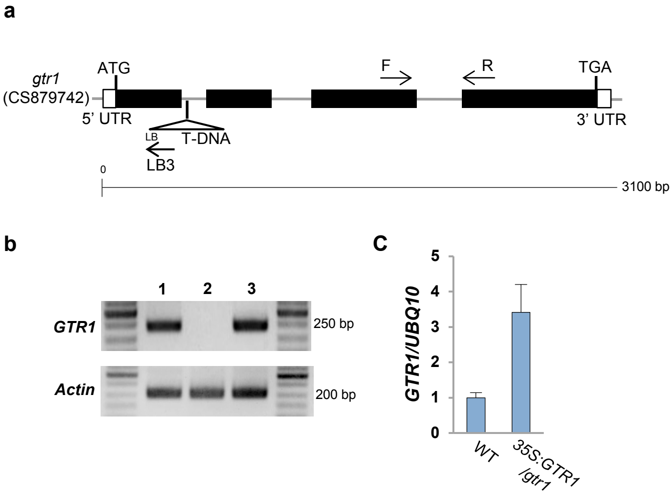

Supplementary Figure 1. Expression analysis of *Arabidopsis* lines used in this study

(a) Schematic map of the T-DNA insertion sites in *gtr1* mutants. The mutant *gtr1* carried a T-DNA insertion in the first intron of *GTR1*. Black boxes denote coding regions; each white box denotes an untranslated region (UTR); F and R, forward and reverse primers used for RT-PCR; LB, left border primer of T-DNA.

(b) *GTR1* expression in mutant lines. Total RNA from 10-day-old seedlings grown in liquid medium of WT (lane 1), *gtr1* (lane 2), and *35S:GTR1/gtr1* (lane 3) was used for RT-PCR. *Actin* was used as an internal control. Both *GTR1* and *Actin* were amplified for 35 cycles. Outer lanes show molecular-weight markers.

(c) Quantitative RT-PCR of *GTR1* transcript levels in WT and *35S:GTR1/gtr1*. Error bars represent ± SD (n = 3). *UBQ10* was used as a reference gene.

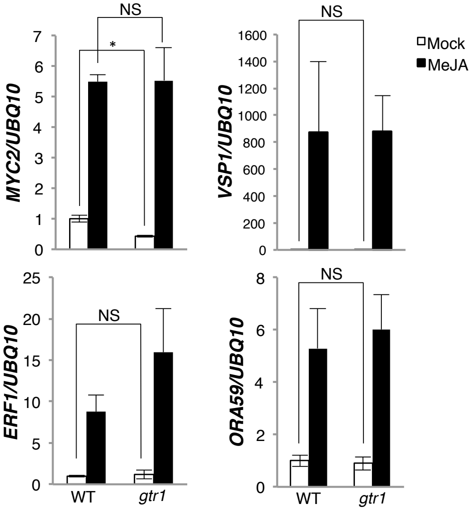

Supplementary Figure 2. Expression of JA-responsive genes in *gtr1* seedlings
Ten-day-old liquid-cultured *Arabidopsis* Columbia WT or *gtr1* seedlings were treated with 0.02% ethanol (white bars) or 20 μM MeJA (black bars) for 24 h (for *MYC2* and *VSP1*) or 0.5 h (for *ERF1* and *ORA59*), followed by quantitative RT-PCR. *UBQ10* was used as a reference gene. Relative gene expression was calculated by normalising to the value of WT treated with 0.02% ethanol. Values are the mean ± SD of three biological replicates. *$p < 0.05$; Tukey-Kramer comparison test.

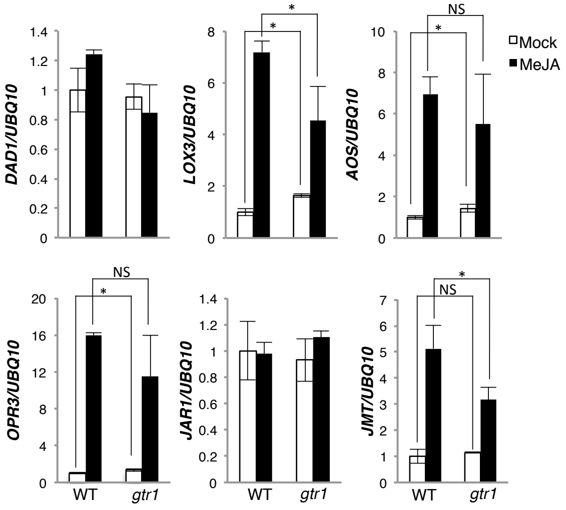

Supplementary Figure 3. Expression of JA biosynthesis genes in *gtr1* seedlings

Ten-day-old liquid-cultured *Arabidopsis* Columbia WT or *gtr1* seedlings were treated with 0.02% ethanol (white bars) or 20 μM MeJA (black bars) for 0.5 h for quantitative RT-PCR. *UBQ10* was used as a reference gene. Relative gene expression was calculated by normalising to the value of WT treated with 0.02% ethanol. Values are the mean ± SD of three biological replicates. *p < 0.05; Tukey-Kramer comparison test.

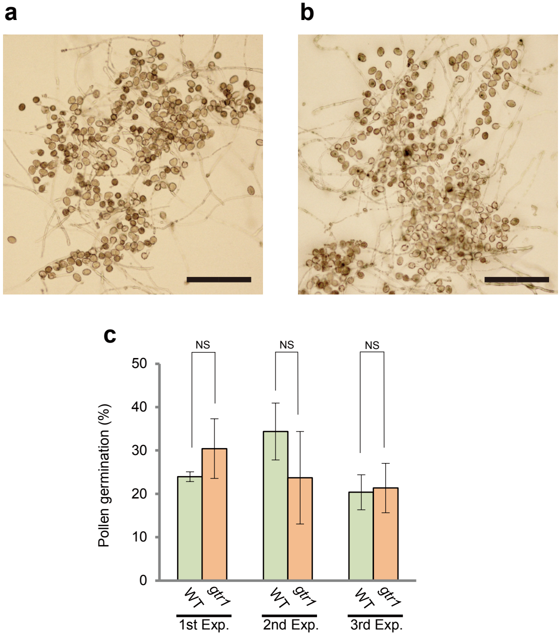

Supplementary Figure 4. *In vitro* pollen germination of WT and *gtr1*

Photographs of pollen grains from WT (a) and *gtr1* (b). Pollen was incubated on germination medium (see Methods) at 23 °C for 24 h. bars = 200 µm

(c) Pollen germination rate *in vitro*. Germination rate of pollen averaged from three independent flowers is presented as the mean ± SD. NS = not significant ($p > 0.05$); Student's t-test.

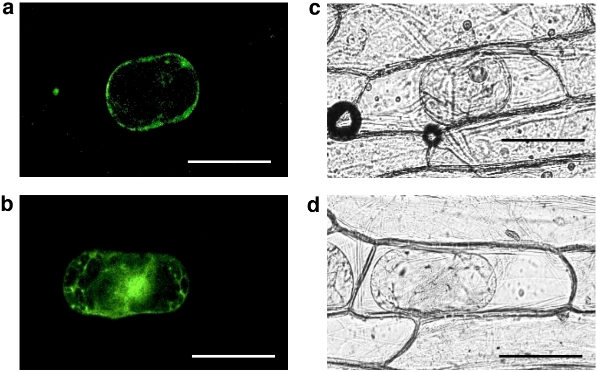

Supplementary Figure 5. Subcellular localisation of GTR1
Onion epidermal cells transiently transformed with either the GTR1-EGFP fusion construct or unfused EGFP were incubated in 0.8 M mannitol to induce plasmolysis and then imaged with confocal microscopy.
(a) Fluorescence image of an epidermal cell expressing the GTR1-EGFP fusion protein and the corresponding bright-field image (c). (b) Fluorescence image of an epidermal cell expressing EGFP as a control and the corresponding bright-field image (d). Bar = 100 µm.

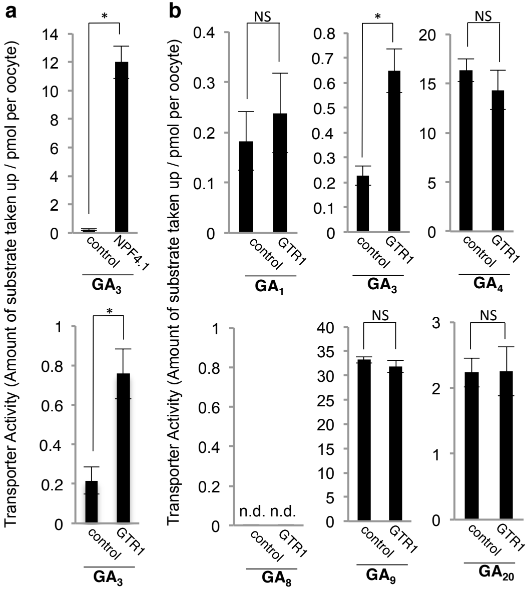

Supplementary Figure 6. NPF4.1- and GTR1-mediated transport of hormones into *Xenopus* oocytes
(a) Twenty-four hours after cRNA injection, kulori-based buffer (pH 5.0) containing 100 μM GA_3 was added to oocytes and incubated at 17 °C for 24 h. Oocytes were washed with sorbitol solution, homogenised in extract medium, and incubated at 4 °C for 24 h. After centrifugation, supernatants were collected. Samples were subjected to UPLC/TOFMS analysis. Control means water injection into *Xenopus* oocytes. Values are the mean ± SD of five biological replicates. $*p < 0.05$; Student's *t*-test.
(b) Twenty-four hours after cRNA injection, kulori-based buffer (pH 5.0) containing 100 μM GA_1, 100 μM GA_3, 100 μM GA_4, 100 μM GA_8, 100 μM GA_9, or 100 μM GA_{20} was added to oocytes respectively and incubated at 17 °C for 24 h. Oocytes were washed with sorbitol solution, homogenised in extract medium, and incubated at 4 °C for 24 h. After centrifugation, supernatants were collected. Samples were subjected to UPLC/TOFMS analysis. Control means water injection into *Xenopus* oocytes. Values are the mean ± SD of five biological replicates. n.d. = not detected. $*p < 0.05$, NS = not significant ($p > 0.05$); Student's *t*-test.

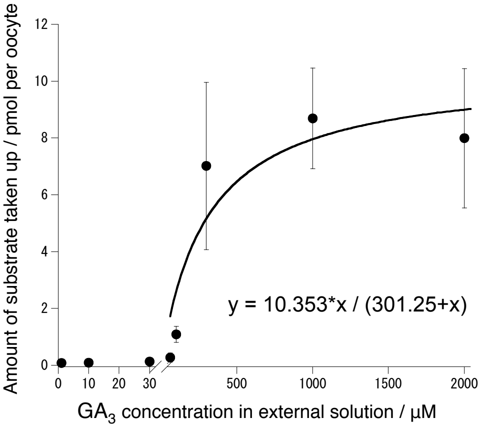

Supplementary Figure 7. Kinetic analysis of GTR1-mediated GA_3 transport
Amount of substrate taken up measured at pH 5.0 is plotted against GA_3 concentration. The saturation curve was best fitted to the Michaelis-Menten equation. Error bars are ± SD, n = 5.

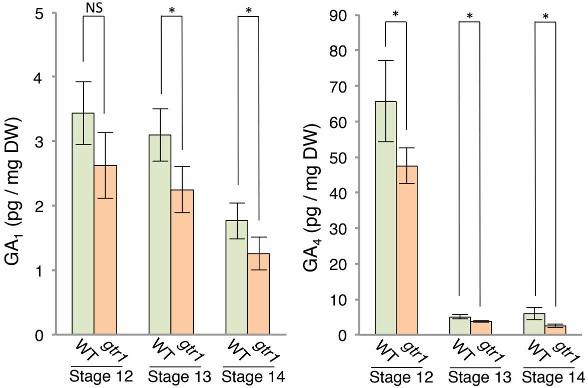

Supplementary Figure 8. Quantification of GA_4 and GA_1 content in *gtr1* flowers
WT and mutant flowers were harvested at the specific stages indicated. Averaged data from four independent biological experiments with error bars (± SD) are presented. *$p < 0.05$, NS = not significant ($p > 0.05$); Student's *t*-test.

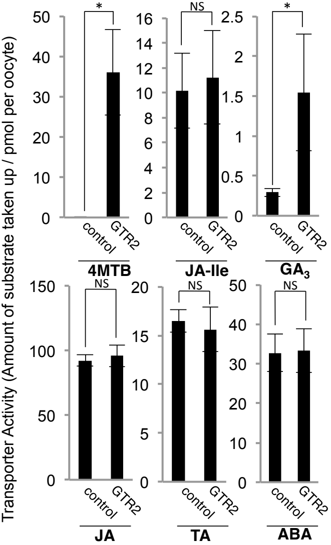

Supplementary Figure 9. GTR2-mediated transport of hormones into *Xenopus* oocytes
Forty-eight hours after cRNA injection, Kulori-based buffer (pH 5.0) containing 100 µM 4MTB, 100 µM JA, 100 µM TA, 100 µM JA-Ile, 100 µM ABA and 100 µM GA_3 was added to oocytes and incubated at 17 °C for 24 h. Oocytes were washed with sorbitol solution, homogenised in extract medium, and incubated at 4 °C for 24 h. After centrifugation, supernatants were collected. Samples were subjected to UPLC/TOFMS analysis. Control means water injection into *Xenopus* oocytes. Values are the mean ± SD of six biological replicates. *p < 0.05, NS = not significant (p > 0.05); Student's *t*-test.

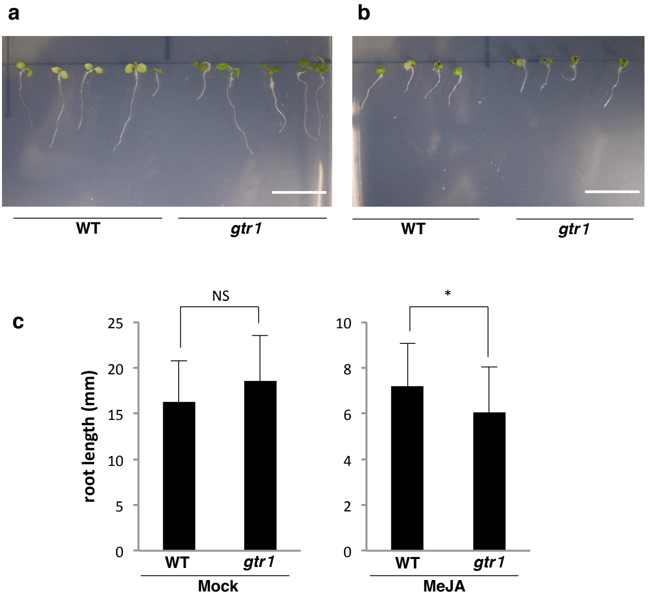

Supplementary Figure 10. Root lengths of WT and *gtr1* seedlings after MeJA treatment
(a) Plants were grown on MS medium containing 1% sucrose and 0.02% ethanol for 7 days. Bar = 10 mm.
(b) Plants were grown on MS medium containing 1% sucrose and 20 µM MeJA for 7 days. Bar = 10 mm.
(c) Root length of WT and *gtr1*. Average primary root length in plants grown on mock (0.02% ethanol) or 20 µM MeJA–containing medium for 7 days are shown as the mean ± SD (n = 10). NS = not significant ($p > 0.05$), *$p < 0.05$; Student's *t*-test.

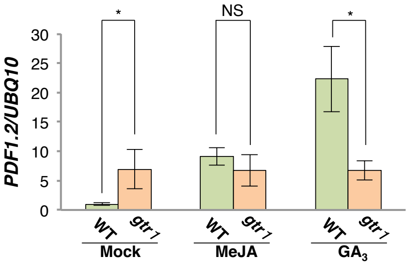

Supplementary Figure 11. Expression of *PDF1.2* in *gtr1* seedlings.
Ten-day-old liquid-cultured *Arabidopsis* Columbia WT or *gtr1* seedlings were treated with 0.02% ethanol (mock), 20 µM MeJA, or 20 µM GA_3 for 24 h, followed by quantitative RT-PCR. *UBQ10* was used as a reference gene. Relative gene expression was calculated by normalising to the value of WT treated with 0.02% ethanol. Values are the mean ± SD of three biological replicates. *$p < 0.05$, NS = not significant ($p > 0.05$); Tukey-Kramer comparison test.

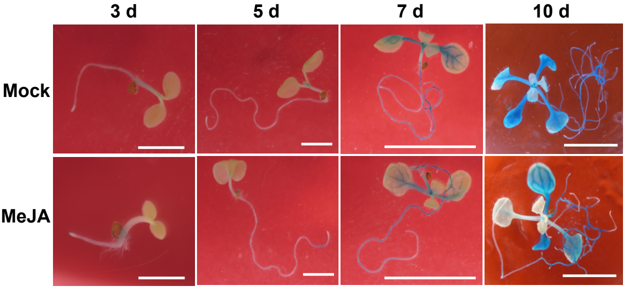

Supplementary Figure 12. Localisation of GTR1
Histochemical analysis of GUS activity in transgenic plants expressing the GUS reporter gene under the control of the GTR1 promoter. Plants were grown on MS medium for the indicated terms before treatment with or without 20 µM MeJA for 3 h. Bar = 1 mm (3 days (d), 5 d) and 10 mm (7 d, 10 d).

Supplementary Table 1-1. List of genes, which co-expressed with JA biosynthetic genes in the top 20 mutual rank of the Pearson's correlation coefficient. Transporters are indicated by boldface. JA biosynthetic genes and relatated genes are indicated by italic face.

Mutual Rank	Locus	annoattion
	**At2g44810**	***DEFECTIVE IN ANTHER DEHISCIENCE 1* (*DAD1*)**
1	At1g62210	unknown protein
2	At1g31670	Copper amine oxidase family protein
3	At3g04170	RmlC-like cupins superfamily protein
4	At3g04200	RmlC-like cupins superfamily protein
5	At3g29260	NAD(P)-binding Rossmann-fold superfamily protein
6	At1g68380	Core-2/I-branching beta-1,6-N-acetylglucosaminyltransferase family protein
7	At1g66720	S-adenosyl-L-methionine-dependent methyltransferases superfamily protein
8	At3g04150	RmlC-like cupins superfamily protein
9	At2g07230	transposable element gene
10	At1g65670	cytochrome P450, family 702, subfamily A, polypeptide 1
11	At4g07750	transposable element gene
**12**	**At1g27080**	**nitrate transporter 1.6 (NRT1.6)**
13	At1g43570	transposable element gene
14	At2g13230	transposable element gene
15	At5g01860	C2H2 and C2HC zinc fingers superfamily protein
16	At5g50750	reversibly glycosylated polypeptide 4
17	At5g56880	unknown protein
18	At5g26640	unknown protein
19	At5g49050	unknown protein
20	At3g24680	transposable element gene

Mutual Rank	Locus	annoattion
	**At1g17420**	***LIPOXYGENASE 3* (*LOX3*)**
1	At1g72520	*LIPOXYGENASE 4* (*LOX4*)
2	At1g17380	*JASMONATE-ZIM-DOMAIN PROTEIN 5* (*JAZ5*)
3	At3g25780	*ALLENE OXIDE CYCLASE 3* (*AOC3*)
4	At1g20510	*OPC-8:0 CoA LIGASE 1* (*OPCL1*)
**5**	**At1g61890**	**MATE efflux family protein**
6	At2g06050	*12-oxo-PHYTODIENOATE REDUCTASE 3* (*OPR3*)
7	At1g73080	PEP1 receptor 1
8	At2g34930	disease resistance family protein / LRR family protein
9	At1g30135	*JASMONATE-ZIM-DOMAIN PROTEIN 8* (*JAZ8*)
10	At5g42650	*ALLENE OXIDE SYNTHASE*
**11**	**At3g47960**	***GLUCOSINOLATE TRANSPORTER 1* (*GTR1*)**
12	At5g13220	*JASMONATE-ZIM-DOMAIN PROTEIN 10* (*JAZ10*)
13	At3g51450	Calcium-dependent phosphotriesterase superfamily protein
14	At2g46510	*ABA-inducible BHLH-type transcription factor/JA-ASSOCIATED MYC2-LIKE1* (*JAM1*)
15	At2g34600	*JASMONATE-ZIM-DOMAIN PROTEIN 7* (*JAZ7*)
16	At3g25180	cytochrome P450, family 82, subfamily G, polypeptide 1
17	At3g48520	cytochrome P450, family 94, subfamily B, polypeptide 3
18	At5g14700	NAD(P)-binding Rossmann-fold superfamily protein
19	At5g54170	Polyketide cyclase/dehydrase and lipid transport superfamily protein
20	At4g34410	redox responsive transcription factor 1

Mutual Rank	Locus	annoattion
	**At1g72520**	***LIPOXYGENASE 4* (*LOX4*)**
1	At1g17420	*LIPOXYGENASE 3* (*LOX3*)
2	At1g20510	*OPC-8:0 CoA LIGASE 1* (*OPCL1*)
3	At1g17380	*JASMONATE-ZIM-DOMAIN PROTEIN 5* (*JAZ5*)
4	At1g80840	WRKY DNA-binding protein 40
5	At3g16860	COBRA-like protein 8 precursor
6	At4g24380	unknown protein
7	At3g25780	*ALLENE OXIDE CYCLASE 3* (*AOC3*)
8	At2g44840	ethylene-responsive element binding factor 13
9	At4g31800	WRKY DNA-binding protein 18
10	At3g02840	ARM repeat superfamily protein
11	At1g28370	ERF domain protein 11
12	At3g01830	Calcium-binding EF-hand family protein
13	At5g14700	NAD(P)-binding Rossmann-fold superfamily protein
14	At2g24600	Ankyrin repeat family protein
15	At5g59730	exocyst subunit exo70 family protein H7
16	At2g32140	transmembrane receptors
17	At5g64870	SPFH/Band 7/PHB domain-containing membrane-associated protein family
18	At1g73080	PEP1 receptor 1
	At5g47910	respiratory burst oxidase homologue D
20	At4g34410	redox responsive transcription factor 1

Supplementary Table 1-2.

Mutual Rank	Locus	annoattion
	**At5g42650**	***ALLENE OXIDE SYNTHASE***
1	At2g06050	*12-oxo-PHYTODIENOATE REDUCTASE 3* (*OPR3*)
**2**	**At5g44050**	**MATE efflux family protein**
3	At1g19670	chlorophyllase 1
4	At4g23600	Tyrosine transaminase family protein
5	At4g24350	Phosphorylase superfamily protein
6	At3g51450	Calcium-dependent phosphotriesterase superfamily protein
7	At1g17420	*LIPOXYGENASE 3* (*LOX3*)
**8**	**At3g47960**	***GLUCOSINOLATE TRANSPORTER 1* (*GTR1*)**
9	At5g58670	phospholipase C1
10	At5g47240	nudix hydrolase homolog 8
11	At1g44350	IAA-leucine resistant (ILR)-like gene 6
12	At1g32640	Basic helix-loop-helix (bHLH) DNA-binding family protein
13	At4g14680	Pseudouridine synthase/archaeosine transglycosylase-like family protein
14	At1g20510	*OPC-8:0 CoA LIGASE 1* (*OPCL1*)
15	At1g72450	*JASMONATE-ZIM-DOMAIN PROTEIN 6* (*JAZ6*)
16	At4g30530	Class I glutamine amidotransferase-like superfamily protein
17	At1g70700	TIFY domain/Divergent CCT motif family protein
18	At3g45140	*LIPOXYGENASE 2* (*LOX2*)
19	At1g52000	Mannose-binding lectin superfamily protein
20	At2g46510	*ABA-inducible BHLH-type transcription factor/JA-ASSOCIATED MYC2-LIKE1* (*JAM1*)

Mutual Rank	Locus	annoattion
	**At3g25780**	***ALLENE OXIDE CYCLASE 3* (*AOC3*)**
1	At1g17380	*JASMONATE-ZIM-DOMAIN PROTEIN 5* (*JAZ5*)
2	At1g19180	*JASMONATE-ZIM-DOMAIN PROTEIN 1* (*JAZ1*)
3	At1g17420	*LIPOXYGENASE 3* (*LOX3*)
4	At1g20510	*OPC-8:0 CoA LIGASE 1* (*OPCL1*)
5	At1g72520	*LIPOXYGENASE 4* (*LOX4*)
6	At2g29440	glutathione S-transferase tau 6
7	At3g23250	myb domain protein 15
8	At1g30135	*JASMONATE-ZIM-DOMAIN PROTEIN 8* (*JAZ8*)
9	At2g06050	*12-oxo-PHYTODIENOATE REDUCTASE 3* (*OPR3*)
10	At2g22500	uncoupling protein 5
11	At2g39420	alpha/beta-Hydrolases superfamily protein
12	At5g13220	*JASMONATE-ZIM-DOMAIN PROTEIN 10* (*JAZ10*)
13	At1g26730	EXS (ERD1/XPR1/SYG1) family protein
14	At3g09940	monodehydroascorbate reductase
15	At3g50760	galacturonosyltransferase-like 2
16	At2g34930	disease resistance family protein / LRR family protein
17	At3g51450	Calcium-dependent phosphotriesterase superfamily protein
18	At2g27690	cytochrome P450, family 94, subfamily C, polypeptide 1
19	At1g06620	2-oxoglutarate (2OG) and Fe(II)-dependent oxygenase superfamily protein
**20**	**At1g61890**	**MATE efflux family protein**

Mutual Rank	Locus	annoattion
	**At1g13280**	***ALLENE OXIDE CYCLASE 4* (*AOC4*)**
1	At5g65020	annexin 2
2	At3g10260	Reticulon family protein
3	At1g66350	RGA-like 1
4	At3g12610	Leucine-rich repeat (LRR) family protein
5	At2g01150	RING-H2 finger protein 2B
6	At5g09980	elicitor peptide 4 precursor
7	At4g12880	early nodulin-like protein 19
8	At5g50375	cyclopropyl isomerase
9	At1g48330	unknown protein
10	At5g50180	Protein kinase superfamily protein
11	At1g54030	GDSL-like Lipase/Acylhydrolase superfamily protein
12	At1g78660	gamma-glutamyl hydrolase 1
13	At5g15230	GAST1 protein homolog 4
14	At2g34470	urease accessory protein G
15	At5g65640	beta HLH protein 93
16	At3g19820	cell elongation protein / DWARF1 / DIMINUTO (DIM)
17	At3g59760	O-acetylserine (thiol) lyase isoform C
18	At1g05620	uridine-ribohydrolase 2
19	At2g47320	Cyclophilin-like peptidyl-prolyl cis-trans isomerase family protein
20	At3g45310	Cysteine proteinases superfamily protein

Supplementary Table 1-3.

Mutual Rank	Locus	annoattion
	**At2g06050**	***12-oxo-PHYTODIENOATE REDUCTASE 3 (OPR3)***
1	At1g20510	*OPC-8:0 CoA LIGASE 1* (*OPCL1*)
2	At3g51450	Calcium-dependent phosphotriesterase superfamily protein
3	At5g42650	*ALLENE OXIDE SYNTHASE*
4	At1g17420	*LIPOXYGENASE 3* (*LOX3*)
5	At1g17380	*JASMONATE-ZIM-DOMAIN PROTEIN 5* (*JAZ5*)
6	At1g32640	Basic helix-loop-helix (bHLH) DNA-binding family protein
7	At3g25780	*ALLENE OXIDE CYCLASE 3* (*AOC3*)
**8**	**At3g47960**	***GLUCOSINOLATE TRANSPORTER 1 (GTR1)***
9	At1g74950	TIFY domain/Divergent CCT motif family protein
10	At1g44350	IAA-leucine resistant (ILR)-like gene 6
11	At5g13220	*JASMONATE-ZIM-DOMAIN PROTEIN 10* (*JAZ10*)
12	At5g47240	nudix hydrolase homolog 8
13	At1g72450	*JASMONATE-ZIM-DOMAIN PROTEIN 6* (*JAZ6*)
14	At5g05600	2-oxoglutarate (2OG) and Fe(II)-dependent oxygenase superfamily protein
15	At2g46510	*ABA-inducible BHLH-type transcription factor/JA-ASSOCIATED MYC2-LIKE1* (*JAM1*)
16	At5g53050	alpha/beta-Hydrolases superfamily protein
17	At2g34930	disease resistance family protein / LRR family protein
18	At1g19180	*JASMONATE-ZIM-DOMAIN PROTEIN 1* (*JAZ1*)
19	At1g70700	TIFY domain/Divergent CCT motif family protein
20	At1g73080	PEP1 receptor 1

Mutual Rank	Locus	annoattion
	**At1g20510**	***OPC-8:0 CoA LIGASE 1 (OPCL1)***
1	At1g72520	*LIPOXYGENASE 4* (*LOX4*)
2	At2g06050	*12-oxo-PHYTODIENOATE REDUCTASE 3* (*OPR3*)
3	At1g17420	*LIPOXYGENASE 3* (*LOX3*)
4	At1g32640	Basic helix-loop-helix (bHLH) DNA-binding family protein
5	At1g17380	*JASMONATE-ZIM-DOMAIN PROTEIN 5* (*JAZ5*)
6	At3g25780	*ALLEME OXIDE CYCLASE 3* (*AOC3*)
7	At1g19180	*JASMONATE-ZIM-DOMAIN PROTEIN 1* (*JAZ1*)
8	At3g51450	Calcium-dependent phosphotriesterase superfamily protein
9	At1g74950	TIFY domain/Divergent CCT motif family protein
10	At5g42650	*ALLENE OXIDE SYNTHASE*
11	At4g14680	Pseudouridine synthase/archaeosine transglycosylase-like family protein
12	At5g47220	ethylene responsive element binding factor 2
13	At1g27770	autoinhibited Ca2+-ATPase 1
14	At2g42760	unknown protein
15	At5g47910	respiratory burst oxidase homologue D
16	At5g59730	exocyst subunit exo70 family protein H7
17	At4g24380	unknown protein
18	At1g72450	*JASMONATE-ZIM-DOMAIN PROTEIN 6* (*JAZ6*)
19	At5g66210	calcium-dependent protein kinase 28
20	At2g46510	*ABA-inducible BHLH-type transcription factor/JA-ASSOCIATED MYC2-LIKE1* (*JAM1*)

Mutual Rank	Locus	annoattion
	**At2g46370**	***JASMONATE RESISTANT 1 (JAR1)***
1	At4g30530	Class I glutamine amidotransferase-like superfamily protein
2	At1g53310	phosphoenolpyruvate carboxylase 1
3	At5g22630	arogenate dehydratase 5
**4**	**At3g47960**	***GLUCOSINOLATE TRANSPORTER 1 (GTR1)***
5	At5g53760	Seven transmembrane MLO family protein
6	At5g12010	unknown protein
7	At4g34230	cinnamyl alcohol dehydrogenase 5
**8**	**At3g54140**	**peptide transporter 1**
9	At5g03630	Pyridine nucleotide-disulphide oxidoreductase family protein
10	At1g02270	Calcium-binding endonuclease/exonuclease/phosphatase family
11	At1g08800	Protein of unknown function, DUF593
12	At4g26080	Protein phosphatase 2C family protein
13	At3g20500	purple acid phosphatase 18
14	At1g74100	sulfotransferase 16
15	At2g46080	BYPASS2
16	At5g60360	aleurain-like protease
17	At3g11660	NDR1/HIN1-like 1
18	At5g52510	SCARECROW-like 8
19	At3g52470	Late embryogenesis abundant (LEA) hydroxyproline-rich glycoprotein family
20	At5g66120	3-dehydroquinate synthase, putative

Supplementary Table 2. Effects of glucosinolate and hormone treatments on silique lengths
Mature silique lengths (mm) were measured. Buds were treated with or without 50 µM 4MTB, GA3 or MeJA as indicated. Values are the mean ± SD of 10 biological replicates. Different letters indicate significant differences ($p < 0.05$). The data were analysed by Tukey-Kramer multiple comparison test.

treatment	mock	4MTB	4MTB + GA_3	4MTB + MeJA	4MTB + MeJA + GA_3
WT	10.50 ± 0.85a	9.40 ± 3.30a	9.20 ± 1.23a	9.40 ± 1.02a	9.40 ± 0.94a
gtr1	3.25 ± 0.63b	3.10 ± 1.5b	9.85 ± 1.06a	3.15 ± 1.38b	9.75 ± 1.55a

Supplementary Table 3. Effects of hormone treatments on seed production
The numbers of mature seeds were counted. Buds were treated with or without 50 μM 4MTB, GA3 or MeJA as indicated. Values are the mean ± SD of 10 biological replicates. Different letters indicate significant differ–ences ($p < 0.05$). The data were analysed by Tukey-Kramer multiple comparison test.

treatment	mock	4MTB	4MTB + GA_3	4MTB + MeJA	4MTB + MeJA + GA_3
WT	40.3 ± 5.9a	38.6 ± 3.3a	34.4 ± 4.0a	38.0 ± 4.3a	34.1 ± 5.2a
gtr1	4.6 ± 4.6b	2.8 ± 3.2b	20.7 ± 7.9c	2.2 ± 3.2b	30.8 ± 7.8d
